# Heme oxygenase-1 determines the cell fate of ferroptotic death of alveolar macrophages in COPD

**DOI:** 10.3389/fimmu.2023.1162087

**Published:** 2023-05-05

**Authors:** Yi Li, Ying Yang, Tingting Guo, Chengxin Weng, Yongfeng Yang, Zhoufeng Wang, Li Zhang, Weimin Li

**Affiliations:** ^1^ Department of Respiratory and Critical Care Medicine, Institute of Respiratory Health, Precision Medicine Key Laboratory, West China Hospital, Sichuan University, Chengdu, China; ^2^ Department of Vascular Surgery, West China Hospital, Sichuan University, Chengdu, China

**Keywords:** chronic obstructive pulmonary disease (COPD), RNA sequencing, alveolar macrophages (AMs), ferroptosis, immune response

## Abstract

**Background:**

Despite an increasing understanding of chronic obstructive pulmonary disease (COPD) pathogenesis, the mechanisms of diverse cell populations in the human lung remain unknown. Using single-cell RNA sequencing (scRNA-Seq), we can reveal changes within individual cell populations in COPD that are important for disease pathogenesis and characteristics.

**Methods:**

We performed scRNA-Seq on lung tissue obtained from donors with non-COPD and mild-to-moderate COPD to identify disease-related genes within different cell types. We testified the findings using qRT−PCR, immunohistochemistry, immunofluorescence and Western blotting from 25 additional subjects and RAW 264.7 macrophages. Targeting ferroptosis with the ferroptosis inhibitor ferrostatin-1, iron chelator deferoxamine or HO-1 inhibitor zinc protoporphyrin was administered in the experimental cigarette smoke COPD mouse model.

**Results:**

We identified two populations of alveolar macrophages (AMs) in the human lung that were dysregulated in COPD patients. We discovered that M2-like AMs modulate susceptibility to ferroptosis by disrupting lipid and iron homeostasis both in vivo and in vitro. The discrepancy in sensitivity to ferroptosis can be determined and regulated by HO-1. In contrast, M1-like AMs showed the ability to attenuate oxidative stress and exert resistance to ferroptosis. In addition, the expression of genes within M2-like AMs is also involved in defects in phagocytosis and lysosome distortion. This ferroptotic phenotype was ameliorated by antiferroptotic compounds, iron chelators and HO-1 inhibitors. During COPD, the accumulation of lipid peroxidation drives ferroptosis-sensitive M2-like AMs, while M1-like AMs show characteristics of ferroptosis resistance. Ferroptotic M2 AMs lose their anti-inflammatory and repair functions but provoke inflammatory responses, resulting in consistent inflammation and tissue damage in the presence of M1 AMs in COPD.

**Conclusion:**

Appropriate interventions in ferroptosis can reduce the occurrence of infections and acute onset, and delay the COPD process.

## Introduction

Iron-dependent cell death known as ferroptosis was initially detected in cancer cells in 2012 by Dixon et al. (1). In the absence of apoptotic hallmarks, this mode of cell death induced by erastin and RSL3 was found to be nonapoptotic and termed. It is characterized by (phospho)lipid peroxidation caused by reactive oxygen species (ROS) during iron-mediated Fenton reactions. Ferroptosis is regulated by glutathione peroxidase 4 (GPX4) by directly converting lipid hydroperoxides (L‐OOH) to nontoxic lipid alcohols (L‐OH) (2). Aberrant regulation of ferroptosis has been implicated in disease pathogenesis and development in the kidney (3), brain (4, 5), liver (6) and lung (7). Recently, protection by ferroptosis inhibitors such as ferrostatin-1 (fer-1) and liproxstatin-1 was also proposed for use in neurodegenerative disease (8–10).

Chronic obstructive pulmonary disease (COPD) is currently the fourth leading cause of morbidity and mortality in the world and is predicted to be the third leading cause of death. It is characterized by chronic airway inflammation, lung destruction and remodeling, resulting in irreversible airflow obstruction (11). Cigarette smoke (CS) exposure is the main risk factor for COPD due to its high concentration of ROS. The consequent cellular oxidative stress provokes inflammation, cell senescence and death. However, although cigarette and other tobacco smoking is the leading environmental risk factor, less than 50% of heavy smokers develop COPD during their lifetime (12, 13). Early studies have demonstrated that accumulated iron and ferritin and increased serum ferritin and nonheme iron were observed in lung epithelial cells and alveolar macrophages during exposure to CS. Recently, a report demonstrated CS-induced ferroptosis in human lung epithelial cells *in vitro* and *in vivo* (14). The experiments revealed the phenomenon of an inverse relationship between ferritin and nuclear receptor coactivator 4 (NCOA4), as well as the negative regulation of GPX4 in epithelial cells. However, the contribution of ferroptosis in other cell types remains unknown. Notably, alveolar macrophages (AMs), which act as innate immune modulators, display a crucial switch in inflammation, cell death and aging, tissue proliferation and repair in COPD pathogenesis. For instance, triggered macrophages amplify the inflammatory process by secreting cytokines and mediators and cause tissue damage by generating and releasing ROS (15, 16). One of the most striking features of AMs in COPD patients is the complexity of the polarization and coexpression of M1 (activated state) and M2 (alternatively activated state) markers (17, 18). In the aspect of functionality, AMs from patients with COPD showed disability in clearing apoptotic cells or bacteria (19, 20), but the nature of this defect in phagocytosis is currently not fully understood. While CS-induced inflammation initiated by the accumulation of lipids in AMs after pulmonary damage has been previously reported (21), a system-wide approach to evaluate AMs in COPD remains to be performed.

The heme oxygenase 1 (HO-1) protein, which is encoded by the *Hmox-1* gene, is known to be an inducible cytoprotective enzyme that copes with oxidative stress. HO-1 catalyzes the first and rate-limiting step in the oxidative degradation of heme to generate biliverdin IXα, carbon monoxide (CO), and ferrous iron (22). Numerous studies have demonstrated that HO-1 and its reaction products can display antioxidant, antiapoptotic, and immunomodulatory effects (23–25). The protective role of HO-1 and CO on inflammation occurs in diseases such as neurodegenerative diseases (26, 27), high-fat induced liver injury and ethanol-induced liver (28, 29), obesity and cardiovascular disease (30), and endothelial injury (31, 32). Deficiency of *Bach1*, a repressor of *Hmox-1*, protected mice from hyperoxic lung injury (33, 34). Studies have reported that the expression of HO-1 is induced in mild COPD compared to smokers without COPD and explained by its potential protective role against ROS-mediated cell senescence and mitochondrial dysfunction. However, this hypothesis has been challenged and not clearly demonstrated. Researchers recently found that HO-1 acts as a critical mediator in ferroptosis induction and plays a causative role in the progression of several diseases; for example, in renal epithelial cells and proximal tubule cells, HO-1 downregulation was associated with ferroptosis, while HO-1 overexpression inhibited ferroptosis (35). On the other hand, overactivation of HO-1 may become detrimental and cytotoxic due to increased intracellular iron, which also induces ferroptosis. The two-sided effect implicates HO-1 in conferring protection or enhancing vulnerability. The complex role of HO-1 in ferroptosis is controversial due to its antiferroptotic or ferroptotic effects *in vitro* and *in vivo*.

In recent years, investigators have used single-cell RNA sequencing technology (scRNA-Seq) to develop an organ-based transcriptomic map of the human body linked to cell populations. The advent of scRNA-Seq allows for the identification of novel and rare cell populations (36, 37) and provides the opportunity to assess the heterogeneity of gene expression in individual cell populations during health and disease (38). Here, in the present study, we report the extensive profiling of whole cells in the lungs by scRNA-Seq of 34572 cells in smokers without COPD and mild-to-moderate COPD patients. We observed a variety of cell types and discovered discrepancies in lipid accumulation and loss of iron homeostasis, leading to peroxidation and ferroptosis in AMs in patients with COPD. We also revealed defects in phagocytosis and lysosome distortion in these cells by analysis of genetic interactions. Our findings reveal an important role of alveolar macrophages in COPD and may guide the design of new strategies for clinical therapeutics aimed at restoring homeostasis.

## Materials and methods

### Human lung tissue collection

Lung resection specimens were obtained from patients who underwent surgery for solitary pulmonary tumors in West China Hospital. Lung tissue was collected at the maximum distance of the tumor. COPD was diagnosed according to the Global Initiative for Chronic Obstructive Lung Disease (GOLD) guidelines before surgery. The patients were divided into three subgroups: 1. Smokers without COPD (n=4), 2. Mild COPD patients (GOLD stage I) (n=4), and 3. Moderate COPD patients (GOLD stage II) (n=4). Clinical information on the patients is shown in **Table S1**. All subjects were enrolled with informed consent from West China Hospital of Sichuan University, China. All patients received surgical treatment, and none of them underwent neoadjuvant therapy before surgery. Cancer clinical stage was matched according to the 8th edition of the American Joint Committee on Cancer (AJCC) TNM staging system. This study was approved by the Ethics Committee of West China Hospital, Sichuan University (project identification code: 2018.270).

### Lung tissue dissociation and single-cell sorting

Lung tissue was transported in Hank’s balanced salt solution (HBSS, Life Technologies) on ice immediately after surgery. Half of the tissue was embedded, and the rest was cut into 1-mm^3^ pieces and digested with collagenase I (2 mg/mL) and IV; (1 mg/mL) in a 15 mL conical tube (BD Falcon) at 37°C for 30 min on a tube revolver (Thermo) with frequent agitation. All samples were then filtered through 70 μm and 40 μm nylon mesh filters (BD Biosciences) and centrifuged at 4°C at 400 x g for 5 min. The cell pellet was suspended in red blood cell lysis buffer, centrifuged and resuspended in PBS with 0.04% FBS. Following dissociation, single-cell suspensions were stained with 7-aminoactinomycin D (7-AAD) in a dark room for 15 min before being analyzed by flow cytometry for live-cell sorting with a MoFloAstrios EQ (Beckman Coulter). Cell suspensions were added to the Master Mix to achieve a final number of 8000 cells per reaction for scRNA-seq.

### ScRNA-Seq library preparation and sequencing

For scRNA-seq, single-cell suspensions were converted to barcoded scRNA-seq libraries using the Chromium Single Cell 3’ Library, Gel Bead & Multiplex Kit and Chip Kit (10x Genomics) following the manufacturer’s instructions. In brief, dissociated single cells were coencapsulated into 3-4 nl droplets together with hydrogel beads carrying barcoding reverse transcription primers. Following reverse transcription, the droplets were taken through the following steps: i) second strand synthesis; ii) linear amplification by *in vitro* transcription (IVT); iii) amplified RNA fragmentation; iv) reverse transcription; and v) PCR. The resulting libraries were sequenced on an Illumina NovaSeq-6000 system and mapped to the human genome using CellRanger (10x Genomics).

### ScRNA-Seq analysis

Raw gene expression matrices generated per sample using CellRanger (version 3.0.0) were combined in R (version 3.6.3) and converted to a Seurat object using the Seurat R package (version 3.0.3.9028). After filtering, the gene expression matrices were normalized to the total cellular read count, original sample identity, and mitochondrial read count using linear regression. To identify marker genes of cell clusters, gene expression was required to be >2.5-fold higher than that in the other clusters. The gene ontology (GO) terms were mapped, and sequences were annotated using the software program Blast2GO. The GO annotation results were plotted by R scripts. Following annotation steps, the studied proteins were blasted against the online Kyoto Encyclopedia of Genes and Genomes (KEGG) database (http://geneontology.org/) to retrieve their KEGG orthology identifications and were subsequently mapped to pathways in KEGG. The protein–protein interaction (PPI) data were retrieved from the IntAct molecular interaction database. The network was then visualized using Cytoscape software (version 3.8.0) (https://cytoscape.org/). The degree of each protein was calculated to evaluate the importance of the protein in the PPI network. The abovementioned analysis was performed by Novogene Bioinformatics Technology Co., Ltd. (Beijing, China).

For validation data, public genomics data in the Gene Expression Omnibus (GEO) database were downloaded. Three datasets (GSE47460-GPL14550, GSE37768 and GSE52509) were used, and differential expression was assessed.

### Histology analysis

Human lung samples were fixed in 4% paraformaldehyde at room temperature for 48 hours. They were dehydrated using a graded ethanol series, immersed in xylene and embedded in paraffin. The samples were cut into 4 μm sections and stained with hematoxylin and eosin (H&E), Masson’s trichrome or Perls’ blue. For immunochemistry and immunofluorescence staining, the sections were blocked with normal goat serum and stained using primary antibodies at 4°C overnight in a wet box. Primary antibodies against the following proteins were used: HO-1 (Abcam, ab52947, 1:200), CD68 (Abcam, ab955, 1:200), FTL (Abcam, ab110017, 1:200), SOD2 (Abcam, ab68155, 1:200), FTH1 (Abcam, ab76972, 1:200), and GPX4 (Abcam, ab125066, 1:100). An Envision kit (DAKO) was used for immunohistochemistry. Secondary antibodies were used for immunofluorescence at room temperature for 1 hour in the dark and counterstained with DAPI (Vector Labs). The images were captured on the respective microscope and evaluated. Immunofluorescence images were captured using an N-SIM-S Super Resolution Microscope (Nikon, Tokyo, Japan). Other images were captured on a NanoZoomer Digital Pathology (NDP) scanning system (Hamamatsu, Hamamatsu City, Japan).

### Flow cytometry analysis

Alveolar macrophages (M1 or M2) were isolated from human lung samples using the markers mentioned above and described elsewhere. Briefly, after cell dissociation, fluorescence staining was performed using the following antibodies: anti-human CD45-FITC (MHCD4501, 1:100, eBiosciences), anti-human CD206-PE (12-2069-42, 1:100, Invitrogen), anti-human CD1c-APC (17-0015-42, 1:100, Invitrogen), anti-human CD11b-PerCP/Cy5.5 (301327, 1:100, Biolegend), and anti-human CD163-PE-CY7 (25-1639-42, 1:100, Invitrogen). Cells were analyzed in flow cytometers, and data were analyzed using FlowJo Software version 10.8.1 (FlowJo, LLC).

### Quantitative reverse transcription polymerase chain reaction

RNA was prepared using TRIzol reagent (Invitrogen, CA, USA) and reverse transcribed to cDNA using an iScript cDNA synthesis kit (Bio-Rad, CA, USA). qRT-PCRs were carried out using the SYBR Green Master mix kit (Bio-Rad) according to the instructions. The housekeeping gene *β-Actin* or *Gapdh* was used as an endogenous internal control, and the results were normalized to those of smokers without COPD. The primer sequences are listed in the **Table S2**.

### Western blot analysis

For Western blot analysis, collected lung tissue was washed in cold PBS, ground and lysed by an electric grinder in RIPA Lysis Buffer (P0013B, Beyotime, Haimen, China) with PMSF (ST506, Beyotime). The protein-transferred polyvinylidene difluoride (PVDF) membrane was probed with the respective antibodies. The immunoreactive protein was detected by a ChemiDoc MP Imaging System (Bio-Rad Laboratories, Hercules, CA, USA). Autoradiographs were quantified using ImageJ software (National Institutes of Health, Bethesda, MD, USA).

### Preparation of cigarette smoke extract

Approximately 30–50 mL of cigarette smoke was drawn into the syringe and bubbled into sterile PBS in 15 mL centrifuge tubes (Thermo Fisher) to prepare cigarette smoke (CS) extract. We used one cigarette for the preparation of 10 mL of solution. To remove insoluble particles, the CS extract solution was filtered (0.22 μm; Merck Millipore) and stored for further use.

### Cell culture

RAW 264.7 macrophages were obtained and cultured at 37°C and 5% CO_2_ in DMEM supplemented with 10% heat-inactivated fetal bovine serum (FBS, HyClone) and 10% penicillin−streptomycin (HyClone). The macrophages were polarized in DMEM containing interferon-γ (IFN-γ, 100 ng/mL, Sigma−Aldrich) for the M1 state or interleukin-4 (IL-4, 20 ng/mL, Sigma−Aldrich) for the M2 state for 48 hours as reported and then used for experiments. Cells were plated in 24-well plates and incubated with CS extract with or without the HO-1 agonist cobalt protoporphyrin (CoPP, 100 mM, Sigma−Aldrich) and the HO-1 antagonist zinc protoporphyrin (ZnPP, 5 mM) for 48 hours. The final DMSO concentration for agent dissolution was less than 0.1%. Cells were stained with Fluoroquench fluorescence stain to assess the live versus dead status of cells. Cell death was monitored by LDH release assay. Quantifications of HO-1, CO, Fe, iNOS and NO production in cells or supernatants were performed using ELISA or biochemistry analysis.

### Mouse cigarette smoke exposure and interventions

Male C57BL/6NCrl mice aged 10 weeks were purchased from Vitalriver Biotechnology (Pinghu, China). All experimental protocols were approved by the Institutional Animal Care and Use Committee (IACUC) and Animal Experiment Center of Sichuan University. All animals were cared for in accordance with the requirements of the Laboratory Animal Welfare Act and amendments thereof. Briefly, mice were exposed twice daily for 1 hour, 5 days a week, to the mainstream smoke of 12 cigarettes using a whole-body exposure system for 4 months. Total particulate matter ranged from 700-900 mg/m^3^. The cotinine and carboxyhaemoglobin levels in cigarette smoke-exposed mice are comparable to those observed in human smokers. Age-matched, air-exposed mice served as nonsmoking controls.

The ferroptosis inducer erastin (MCE, 10 mg/kg), ferroptosis inhibitor ferrostatin-1 (MCE, 5 mg/kg), HO-1 inhibitor ZnPP (MCE, 25 mg/kg), and iron chelator deferoxamine (Sigma−Aldrich, 100 mg/kg) were administered intraperitoneally in 125 μL of corn oil once a week. The final DMSO concentration for agent dissolution was less than 0.1%. Sham mice received corn oil with 0.1% DMSO solution only. Mice were sacrificed after the last administration.

For bronchoalveolar lavage (BAL) acquirement, the mouse tracheae were cannulated and lavaged three times with 500 μL of cold PBS after anesthetization. Cells were pelleted, and the cell-free BAL fluid (BALF) was collected and detected.

### Lung function measurements

Mice were anesthetized, tracheostomized and ventilated using a flexiVent system running Flexiware v.7.6.4 software to measure respiratory functions such as forced vital capacities (FVC), forced expiratory volume (FEV), and inspiratory capacity (IC). The FEV/FVC was calculated.

### C11-BODIPY staining

Lungs were harvested, digested and cultured for 2 days to isolate alveolar macrophages to quantify lipid peroxidation. Cells were stained using a solution of C11-BODIPY 581/591 (1 μM; Thermo Fisher) in PBS (Invitrogen, 10 μM) for 1 hour at 37 °C in a tissue culture incubator. After two PBS washes, representative samples were imaged by confocal microscopy (Nikon).

### Mitochondrial membrane potential detection

The cells were digested, resuspended and stained with JC-1 working solution (Beyotime, 10 μg/mL). After mixing, the cells were incubated at 37 °C for 20 minutes. After incubation, the cells were centrifuged, washed, resuspended in JC-1 staining buffer, and then analyzed by flow cytometry. The ratio of the mean fluorescence intensity (MFI) of FL2 (J-aggregates, red)/FL1 (monomer, green) fluorescence was calculated for each sample.

### Statistical analysis

All data were analyzed using SPSS 25.0 and organized using GraphPad Prism 9. Data are presented as the means ± standard errors of the means (SEM). One-way analysis of variance (ANOVA) was used for multiple comparisons, and Dunnett’s test was used for each two-group comparison. At least three parallel experiments were conducted using different samples. A level of *p*<0.05 was accepted as significant.

## Results

### A comprehensive map of the cell types in the lungs of smokers without COPD and individuals with mild-to-moderate COPD

**Figure 1:**
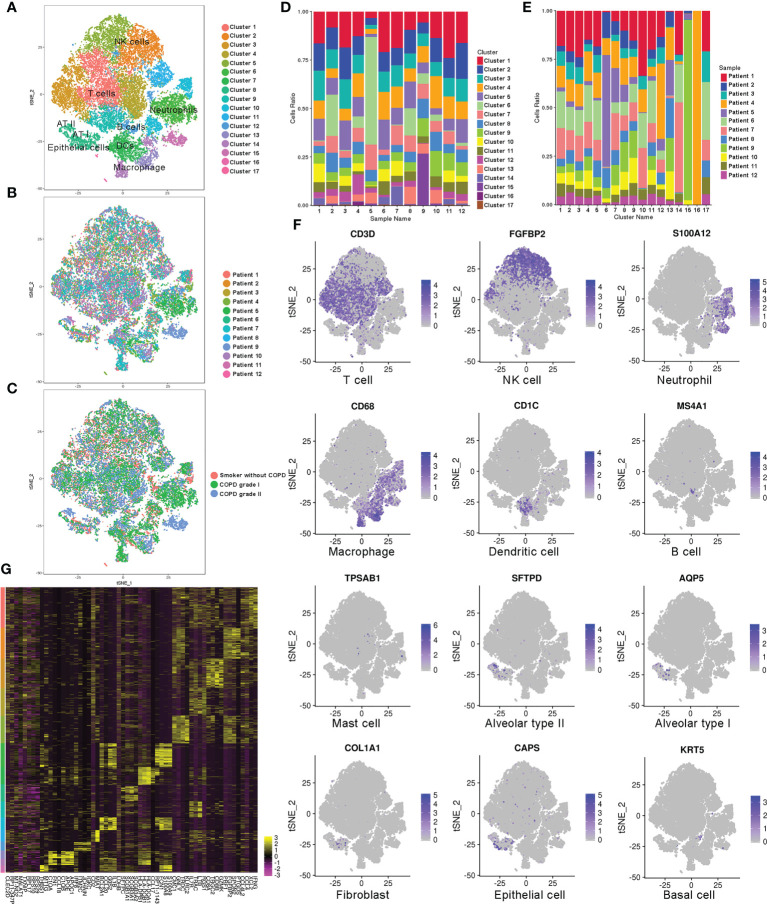
Single-cell RNA-Seq analysis of patients with COPD identifies diverse lung cell populations. **(A)** Cellular populations were identified and visualized using a t-distributed Stochastic Neighbor Embedding (tSNE) plot. **(B)** Each population included cells from smokers without COPD and patients with COPD. **(C)** Cells were grouped as originating from smokers without COPD, mild COPD (COPD grade I) and moderate COPD (COPD grade II). **(D)** Cell type distribution in each sample. **(E)** Cell source in each cluster. **(F)** Classic cell markers were used to label clusters by cell identity as represented in the tSNE plot. **(G)** Heatmap representing gene signatures in each cellular population.

To understand the alteration of immune and nonimmune cell types and states for mild-to-moderate COPD compared to disease-free patients, we collected single-cell profiles from 12 samples according to the grading of illness to construct the lung cellular map (non-COPD; mild-COPD; moderate-COPD: n=4 each) (**Figures 1A−C**, **Supplementary Table S1**). The composition of cells undergoes dramatic alteration along with the inflammatory process and tissue destruction. The pulmonary profile was composed of 56.3%, 71.3% and 65.6% CD45+ immune cells in smokers without COPD and in those with mild and moderate COPD, respectively. Among the immune cells, the percentages of dendritic cells (DCs), B cells and specific subtypes of natural killer (NK) cells declined. Meanwhile, the composition of AMs showed a steady increase in the myeloid cell compartment, which was 3.0% in the non-COPD samples and reached up to 3.8% and 6.4% in mild and moderate COPD, respectively (**Figures 1D, E**). In the CD45+ immune compartment, lymphoid lineages were detected, including T cells (characterized by high expression of *Cd3d*), NK cells (*Fgfbp2*), and B cells (*Ms4a1*), while myeloid cells were separated into neutrophils (*S100a12*), macrophages (*Cd68*), DCs (*Cd1c*), and mast cells (*Tpsab1*). In the nonimmune compartment, alveolar cells type I and II (AT I and II) (*Aqp5* and *Sftpd*), epithelium (*Caps*), club cells (*Scgb3a2*), fibroblasts (*Col1a1*), and basal cells (*Krt5*) were recognized (**Figure 1F**). A two-dimensional representation of immune and nonimmune single cells revealed the separation of cells into diverse lineages (**Figure 1G**).

### Distinct intergroup genes are localized in alveolar macrophages

**Figure 2:**
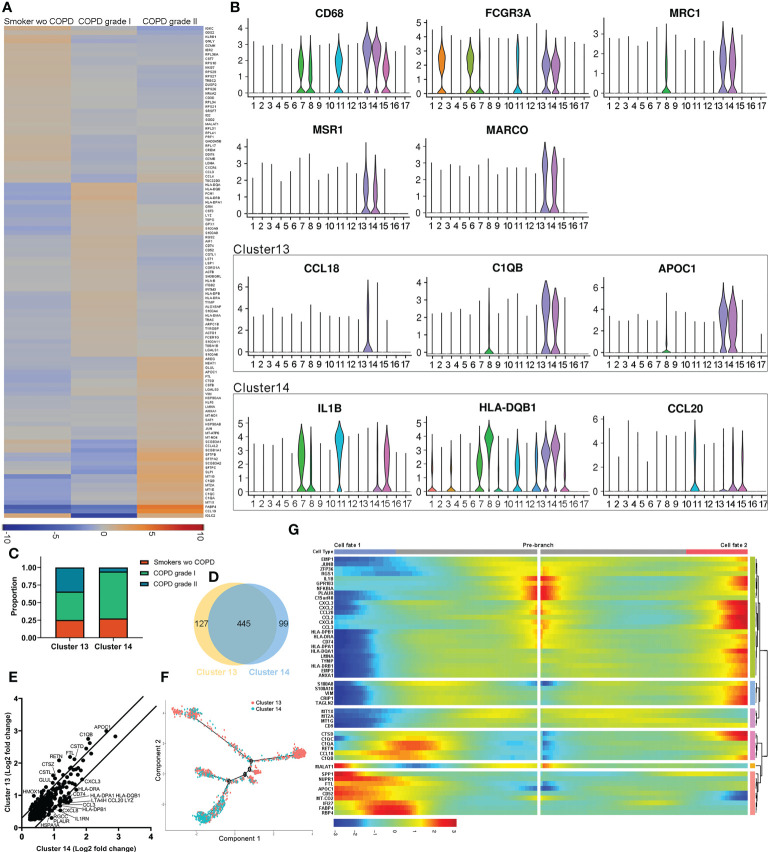
Distinct populations of alveolar macrophages in healthy smoker and COPD lungs. **(A)** Heatmap of significantly differentially expressed genes between smokers without COPD, mild COPD patients and moderate COPD patients. **(B)** Expression of markers and significant differentially expressed genes associated with activated M1 and alternatively activated M2 alveolar macrophages. **(C)** Relative cell contributions from healthy and COPD lungs to each alveolar macrophage cluster. **(D)** Venn diagram of all detected genes in each alveolar macrophage cluster. **(E)** Differential expression of shared genes in each alveolar macrophage cluster. **(F)** Trajectory analysis of the alveolar macrophage state transition in two-dimensional state-space. **(G)** Heatmap showing differentially expressed genes arranged in pseudotemporal patterns.

We analyzed the differentially expressed genes among groups. Transcriptionally, the COPD samples expressed high or elevated levels of *Fabp4*, *Ccl18*, *C1qa*, *C1qb, C1qc, Lmna, Lgals3, Ctsd, Ftl Apoc1, and Glul*, especially in moderate patients. In contrast, the expression levels of *Igkc*, *Klrb1*, *Gnly*, *Gzmh*, *Gzmb*, *Cst7*, *Nkg7*, and *Prf1* were significantly downregulated compared to those in non-COPDs. The profiles demonstrated that the downregulation of key genes in B cells and NKs of COPD patients may be related to susceptibility to infection (**Figure 2A**). Notably, differentially expressed genes were uniquely or partially localized in cells expressing the macrophage hallmarks *Cd68*, *Fcgr3a, Mrc-1* (*Cd206*), Msr-1 and *Marco*. AM-CLST13 cells expressing higher levels of *Ccl18*, *C1qb* and *Apoc1* were classified as M2 alternatively activated macrophages with anti-inflammatory effects, while AM-CLST14 cells expressing higher levels of *Il-18*, *Hla-dqb1* and *Ccl20* were classified as M1 classically activated macrophages with proinflammatory effects (**Figure 2B**, **Supplementary Table S3**). AM-CLST13 increased in the COPD samples; in contrast, AM-CLST14 originated largely from the lungs of patients without or with mild COPD (**Figure 2C**). Our analysis showed 445 identical genes shared in the two AM clusters, as well as 127 and 99 specific genes in each cluster (**Figure 2D**). AM-CLST13 was characterized by high expression of complement factors (*C1qa*, *C1qb* and *C1qc*), genes associated with cathepsin *(Ctsd*, *Ctsl*, *Ctsz)*, ferroptosis (*Ftl*, *Glul*, *Homx-1*) and lipid metabolism (*Apoc1*, *Fabp5*), while AM-CLST14 was characterized by the expression of MHC class II molecules (*Hla-dr*, *Hla-dq* and *Hla-dp*), antigen processing and presentation (*Cd74*) and antioxidant metabolism (*Sod2*) (**Figure 2E**). We performed pseudotime analysis and tracked the gene expression changes along the trajectory of two alveolar macrophage clusters from smokers without COPD and patients with mild and moderate COPD. AM-CLST13 was positioned at the opposite end of AM-CLST14, especially in moderate cases (**Figures 2F**, **S1**). We then identified genes expressed in prebranch, cell fate 1 and cell fate 2 and classified differentially expressed genes into six subsets. The expression profile of subset 1 showed high levels of chemokines, while subset 2 expressed the S100 family of proteins, indicating activation and chemotaxis of the immune system. Subset 3 showed expression of metallothionein, which was consistent with a recent report (39). Subset 4 expressed immunoregulatory genes associated with the complement system, whereas subset 5 was involved in cell cycle regulation and was downregulated in mild and moderate COPD patients. Subset 6, on the other hand, was involved in the regulation of cytosolic ion concentration and stress-induced cell death (**Figures 2G**, **S1**).

### Different cell fates of ferroptotic death of alveolar macrophages

**Figure 3:**
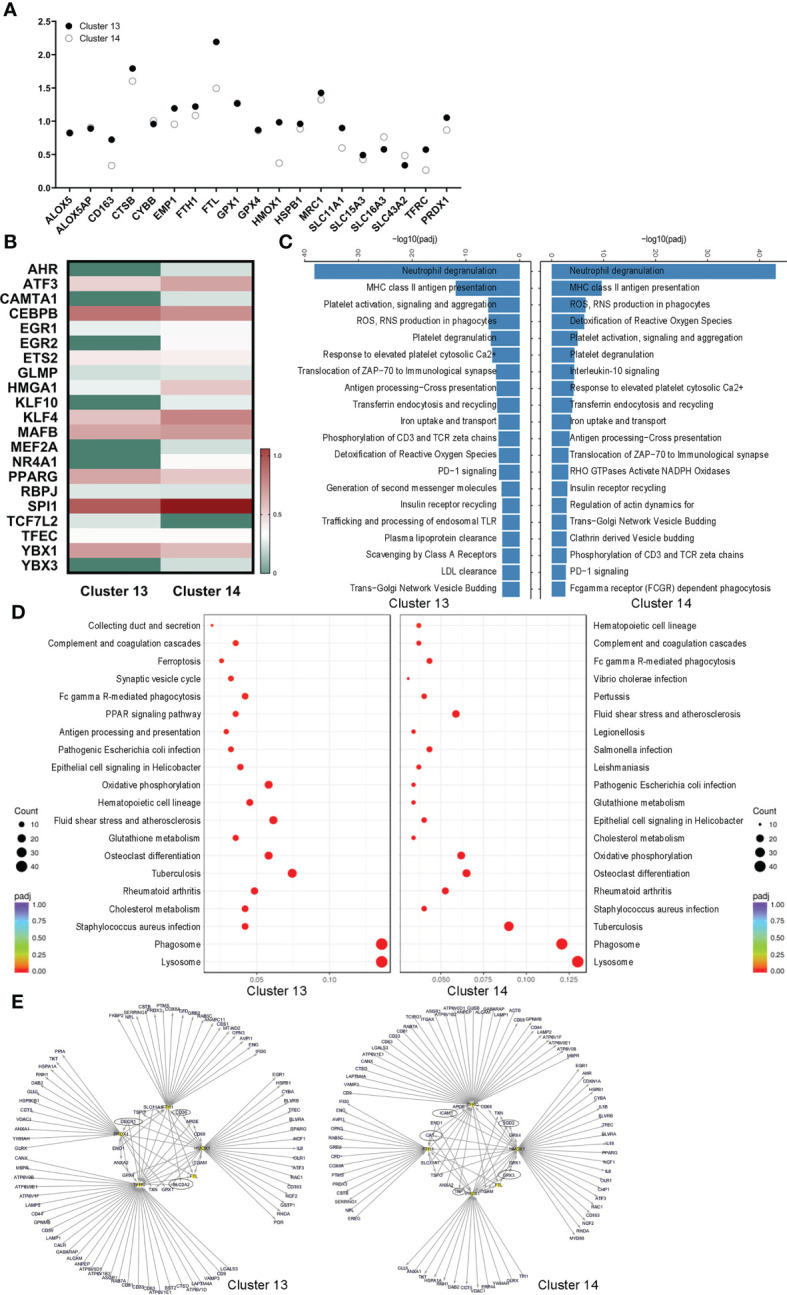
Different cell fates of ferroptotic death of alveolar macrophages. **(A)** Selected ferroptotic genes, **(B)** differential expression of transcription factors, **(C)** Reactome analysis, **(D)** Kyoto Encyclopedia of Genes and Genomes (KEGG) pathway analysis, and **(E)** protein−protein interaction (PPI) networks revealed different cell fates for ferroptotic death between activated M1 and alternatively activated M2 alveolar macrophages in the lungs.

The coexpressed ferroptotic-specific genes were selected and compared between the two alveolar macrophage clusters. Noticeably, the genetic phenotypes of *Emp1*, *Fth1*, *Ftl, Hmox-1*, *Slc11a1*, *Tfrc* and *Prdx1* were different (**Figure 3A**). In addition, the ferroptosis-mediated genes *Acsl1*, *Scd1* and *Por* were uniquely expressed in AM-CLST13 cells (**Table S3**). Meanwhile, the ferroptotic-associated transcription factor expression levels were also distinct, including those of Ahr, Atf3, Cebpb, Egr1, Nr4a1, and Pparg (**Figure 3B**). We then performed GO, KEGG and Reactome analyses. Reactome analysis revealed that AM-CLST13 is involved in the distinctive metabolic networks of plasma lipoprotein and LDL clearance, generation of second messenger molecules, trafficking and processing of endosomal TLRs and scavenging by class A receptors, while AM-CLST14 is involved in IL-10 signaling, RHO GTPases activation of NADPH oxidases, regulation of actin dynamics for phagocytic cup formation, clathrin-derived vesicle budding, and FCGR-dependent phagocytosis (**Figure 3C**). Similarly, KEGG pathways identified that the expression of AM-CLST13 was distinctly enriched in antigen processing and presentation, the PPAR signaling pathway and ferroptosis (**Figure 3D**). Through PPI analysis, the expression profiles of alveolar macrophage clusters and their networks were revealed. Specifically, expressed genes, such as *Decr1*, *Cd36* and *Slc3a2* in AM-CLST13, while *Sod2*, *Gpx3* and *Cat* in AM-CLST14, were also involved in ferroptotic death and resistance, aside from the others we mentioned above. Moreover, most of them were regulated by Hmox-1 (**Figure 3E**).

### Characterization and identification of alveolar macrophages across different stages

**Figure 4:**
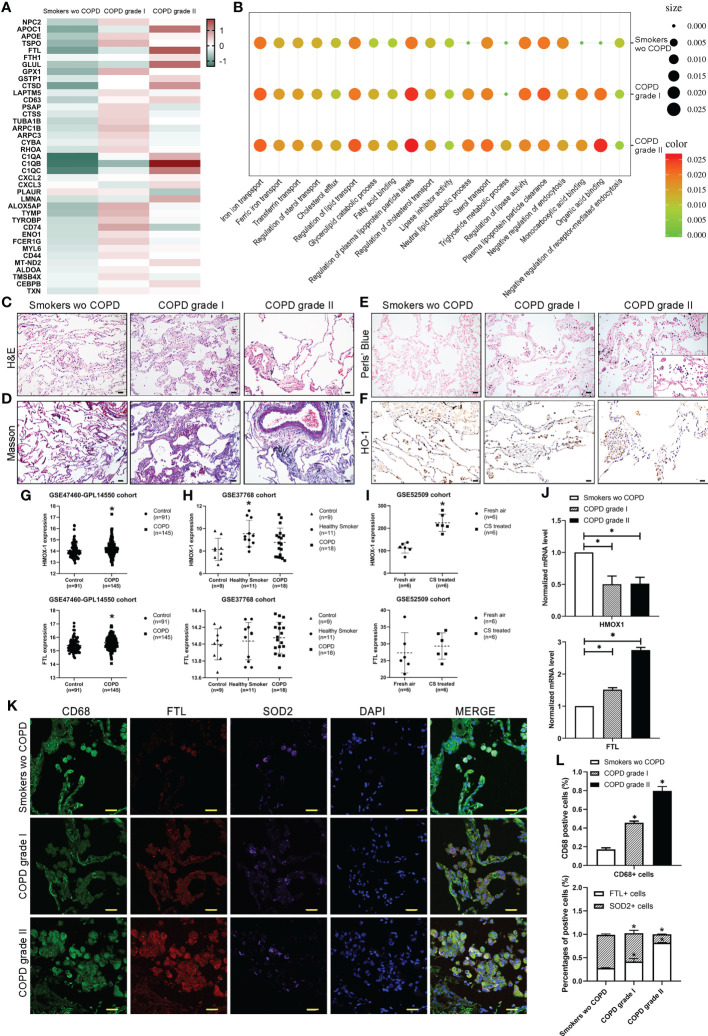
Characterization of macrophages across different stages. **(A)** The expression of selected significant differentially expressed genes across different stages. **(B)** Gene Ontology (GO) enrichment plot of iron and lipid metabolism in COPD. **(C)** H&E and **(D)** Masson’s trichrome staining revealed the morphology of COPD lungs. **(E)** Perls’ staining revealed free iron accumulation in additional COPD samples. **(F)** Immunohistochemical staining for HO-1 showed the expression of HO-1 in COPD patients compared to smokers without COPD. **(G)** The mRNA expression levels of HMOX-1 and FTL in publicly available array data from lung tissue (GSE47460-GPL14550) of healthy subjects (*n* = 91) versus patients with COPD (*n* = 145) **(H)** versus lung tissue (GSE37768) of healthy subjects (*n* = 9) versus heathy smokers (n=11) and patients with COPD (*n* = 18) **(I)** in whole lung from B6 mice exposed to filtered air (*n* = 6) or cigarette smoke (*n* = 8). **(J)** The mRNA expression levels of HMOX-1 and FTL determined by qPCR in whole lungs from our samples. **(K)** Coimmunofluorescence staining for macrophages (CD68, green), activated M1 (FTL, red) and alternatively activated M2 (SOD2, purple) across lung tissues. **(L)** The number and percentage of positive cells. *p<0.05 versus the control group. Scale bar=50 μm.

Heatmaps for representative differentially expressed genes involved in iron transport and metabolism, lipid metabolism, and biosynthetic and catabolic processes from observed alveolar macrophage populations in the three groups are shown and dysregulated (**Figure 4A**). Through Gene Ontology (GO) analysis, the identified transcripts were found to be involved in iron homeostasis, lipid metabolism, some biosynthetic and catabolic processes and endocytosis. Upregulated terms such as “iron ion transport”, “transferrin transport”, “regulation of sterol transport”, “cholesterol efflux” and “triglyceride metabolic process” and downregulated terms such as “lipase inhibitor activity”, “plasma lipoprotein clearance”, and “negative regulation of endocytosis” were observed in M2 alveolar macrophages in COPD lungs (**Figure 4B**). We then examined paraffin-embedded lung tissues from patients. Histologic examination of H&E and Masson’s trichrome staining revealed destruction of alveolar walls with diminished alveolar capillaries, leading to enlarged air spaces. Ongoing inflammation and black pigment are present in advanced disease (**Figures 4C, D**). Perls’ staining revealed that AMs and alveolar epithelial cells in COPD patients had higher levels of free iron accumulation than those in smokers without COPD (**Figure 4E**). The expression of HO-1 (encoded by the *Hmox-1* gene) was decreased in mild and moderate COPD compared to healthy smoker lungs using immunohistochemistry staining (**Figure 4F**). Analysis of an RNA sequencing (RNA-seq) dataset from the public GEO database showed upregulation of *Hmox-1* in COPD compared to healthy controls (**Figures 4G**, **S2**) but slight downregulation compared to smokers without COPD (**Figures 4H**, **S2**). Similarly, analysis based on mice revealed that *Hmox-1* was much more highly expressed in lung tissues from mice treated with CS than in normal tissues from mice treated with filtered air (**Figures 4I**, **S2**). Meanwhile, upregulation of *Ftl* in COPD compared to healthy controls and smokers without COPD was observed in humans and was more highly expressed in lung tissues from mice treated with CS, but the difference was not statistically significant (**Figures 4G−I**). We further explored *Hmox-1* and *Ftl* expression in our clinical samples and found that they were consistent with the data (**Figure 4J**). We then performed coimmunofluorescence staining for macrophages (CD68) and divided them into AM-CLST13 (FTL) and AM-CLST14 (SOD2) based on their expression in tissues from COPD patients and smokers without COPD (**Figure 4K**). According to the cell count, the CD68+ populations increased as COPD progressed. The number and percentage of FTL+ populations increased, while the percentage of SOD2+ populations decreased (**Figure 4L**).

### Profiling of alternatively activated (M2) macrophages in lungs

**Figure 5:**
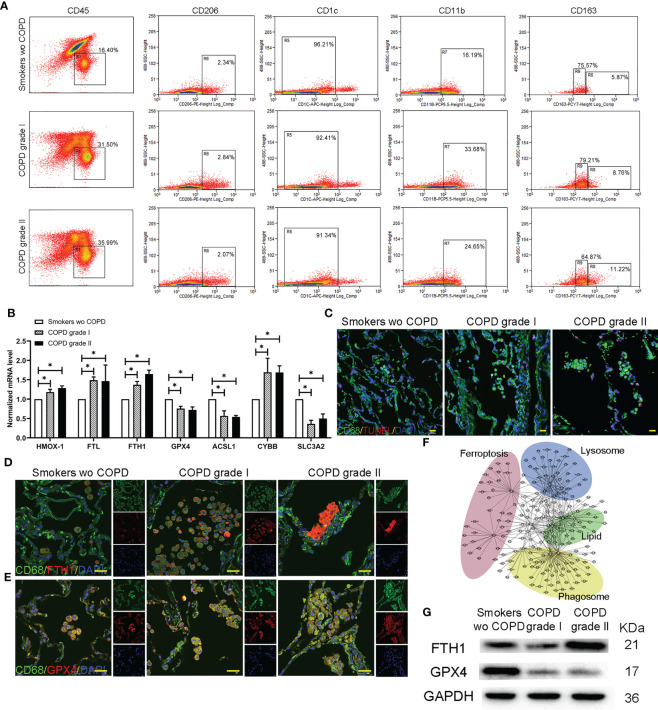
Profiling of alternatively activated (M2) macrophages in lungs. **(A)** Flow cytometry analysis of single-cell suspensions for myeloid cells from whole lungs in smokers without COPD and COPD patients. **(B)** The mRNA levels of ferroptotic genes expressed in separated alternatively activated (M2) macrophages across groups determined by qPCR. **(C)** TUNEL-positive alveolar macrophages indicated that cell death was increased in COPD lungs. Immunofluorescence staining for **(D)** FTH and **(E)** GPX4 in alternatively activated (M2) macrophages in the lungs. **(F)** Gene−gene interaction maps of alveolar macrophages involved in phagosome, lysosome, fat and cholesterol metabolism and ferroptosis are shown. **(G)** Western blotting of FTH and GPX4 was performed to examine alternatively activated (M2) macrophages. *p<0.05 versus the control group. Scale bar=50 μm.

The leukocytes (CD45^+^) were gated with CD206^+^, CD1c^-^ and CD11b^+^ and then segregated into CD163^high^ (R8, AM-CLST13) and CD163 ^intermediate^ (R9, AM-CLST14) by flow cytometry. Consistent with the results, the number and percentage of M2 macrophages increased from 5.87% in smokers without COPD to 8.76% and 11.22% in mild and moderate COPD, respectively. Meanwhile, the percentage of M1 macrophages fluctuated as 75.57%, 79.21% and 64.87% in smokers without COPD and mild and moderate COPD, respectively (**Figure 5A**). Increases in intracellular ferritin (*Fth1*, *Ftl*) and *Cybb*, as well as decreases in *Acsl1*, *Slc3a2* and *Gpx4*, were observed in AM-CLST13 cells (R8) sorted by FACS from mild-moderate COPD patients compared to smokers without COPD, indicating that M2 alveolar macrophages had marked changes in ferroptosis (**Figure 5B**). The cell death TUNEL assay revealed that positive alveolar macrophages were increased in COPD lungs (**Figure 5C**). Immunofluorescence staining showed enhanced FTL and FTH1 expression and reduced expression of GPX4 in alveolar macrophages corresponding to patients’ pathological status (**Figures 4K**, **5D, E**). A protein−protein interaction (PPI) network was constructed, and a specific network was visualized by Cytoscape. The AM-CLST13 networks interacted with phagosome, lysosome, fat and cholesterol metabolism and ferroptosis proteins (**Figure 5F**). Western blotting confirmed these results and revealed the promotion of ferroptosis in AM-CLST13 cells from patients with mild and moderate COPD (**Figure 5G**).

### Heme oxygenase 1 determines sensitivity to ferroptosis in macrophages

**Figure 6:**
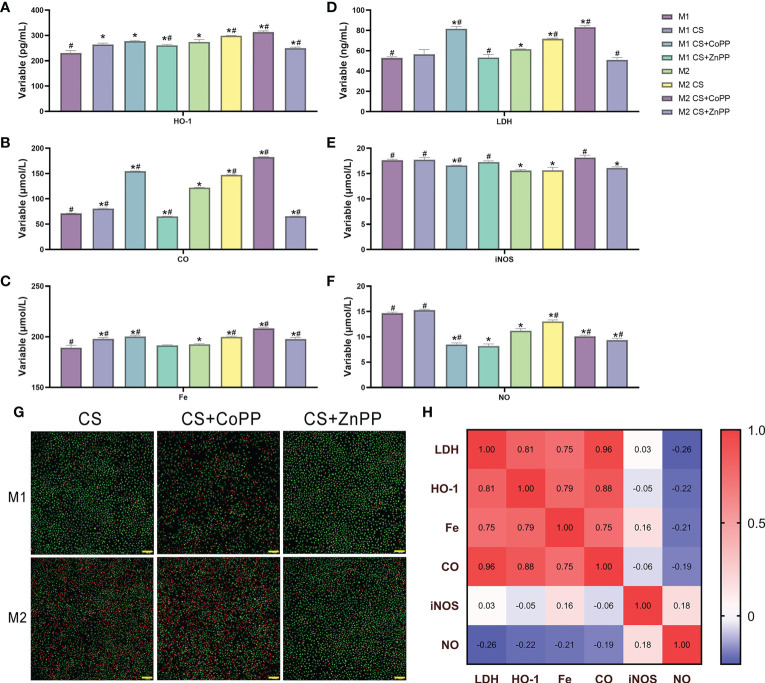
HO-1 determines sensitivity to ferroptosis in RAW 264.7 macrophages. Activated (M1) macrophages display resistance to cigarette smoke-induced ferroptosis compared to alternatively activated (M2) macrophages. RAW 264.7 macrophages were treated with cigarette smoke in the presence or absence of the HO-1 agonist CoPP and antagonist ZnPP. **(A)** Heme oxygenase-1 (HO-1), **(B)** carbon monoxide (CO), **(C)** Fe(II), **(D)** lactate dehydrogenase (LDH), **(E)** inducible nitric oxide synthase (iNOS), and **(F)** nitric oxide (NO) concentrations were examined. **(G)** Apoptosis assay of macrophages using acridine orange/ethidium bromide (AO/EB) staining. **(H)** Correlation coefficient of gene expression levels between HO-1/CO/Fe(II) and iNOS/NO in macrophages. *p<0.05 versus the M1 group; # *p*<0.05 versus the M2 group. Scale bar=100 μm.

To investigate the regulatory mechanism of HO-1 and different cell fates in ferroptosis in alveolar macrophages, we established an exposure protocol for RAW 264.7 macrophages to CS extract and nontoxic concentrations of the HO-1 agonist CoPP or antagonist ZnPP. The experiments revealed that M1 classically activated macrophages exerted high resistance to CS-induced ferroptosis, whereas M2 alternatively activated macrophages were vulnerable. The contents of HO-1, CO and Fe were higher in the M2 versus M1 state (**Figures 6A−C**) and consistently responded with cell death estimated by LDH release (**Figure 6D**). This was effectively preventable by ZnPP. Considering the contribution of iNOS/NO previously reported, our results showed that iNOS/NO was enriched in M1 macrophages, but the changes in iNOS/NO were not significantly different in parallel macrophages (**Figures 6E, F**). We found that the number of M1 macrophages was lower than the number of M2 macrophages, and cells treated with CS extract exhibited higher levels of death in M2 macrophages. Cells treated with CoPP showed increased cell death, whereas ZnPP abolished CS extract-mediated death (**Figure 6G**). There was also a positive correlation between HO-1, CO, and Fe concentrations and LDH levels (Pearson<0.0001,<0.001,<0.0001, respectively) (**Figure 6H**).

### Ferroptosis as a target for protection of lung function in cigarette smoke-induced COPD mice

**Figure 7:**
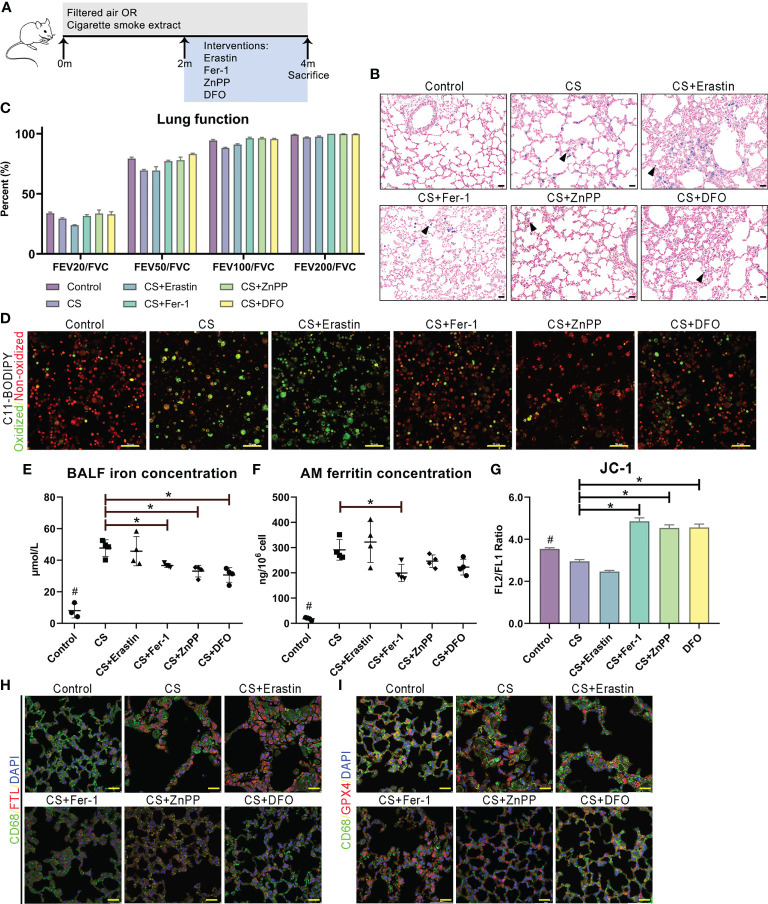
Ferroptosis as a target for protection for lung function in cigarette smoke induced COPD. **(A)** Schematic representation of the ferroptosis treatment protocol in cigarette smoke-treated mice. **(B)** Representative images of lung sections stained with Perls’ stain. **(C)** Lung function data from mice exposed to filtered air or cigarette smoke and treated with erastin (ferroptosis agonist), Fer-1 (ferroptosis antagonist), ZnPP (HO-1 antagonist) and DFO (iron chelator). **(D)** C11-BODIPY581/591 staining showed lipid peroxidation and antioxidant efficacy in membrane systems in alveolar macrophages. **(E)** Fe(II) concentrations in bronchoalveolar lavage fluid. **(F)** Ferritin concentrations released from separated alveolar macrophages. **(G)** Representative images of JC-1 fluorescence in cells exposed to filtered air or cigarette smoke and treated with erastin, Fer-1, ZnPP and DFO. Immunofluorescence analyses of **(H)** FTL and **(I)** GPX4 and CD68 double-positive alveolar macrophages in model mice. *p<0.05 versus the control group. Scale bar=50 μm. # p<0.05 all groups versus the control group.

We examined whether the administration of ferroptosis attenuated lung inflammation, destruction and remodeling in COPD mice by utilizing the ferroptosis inducer erastin, ferroptosis inhibitor Fer-1, HO-1 inhibitor ZnPP, and iron chelator DFO (**Figure 7A**). We observed that the cellular profile and appearance of AMs from the lungs of administered mice were similar to those in humans, and alveolar space enlargement, AM recruitment and iron accumulation in the airway were observed by Perl’s staining (**Figure 7B**). Lung function measurements of mice demonstrated a decreased FEV/FVC ratio, indicating deteriorated expiratory flow and lung compliance (**Figure 7C**). C11-BODIPY581/591 staining showed that lipid peroxidation in isolated alveolar macrophages from mice was attenuated by the administration of Fer-1, ZnPP and DFO (**Figure 7D**). Fe(II) concentrations in bronchoalveolar lavage fluid and ferritin concentrations released from separated alveolar macrophages from mouse lungs were also decreased at different levels (**Figures 7E, F**). Significant increases in mitochondrial membrane potential (measured by JC-1 fluorescence) were observed after treatment with Fer-1, ZnPP and DFO in mouse alveolar macrophages (**Figure 7G**). Immunofluorescence analyses of FTL and GPX4 and CD68 double-positive alveolar macrophages in mice were in accordance with the pathological characteristics of ferroptosis (**Figures 7H, I**). Taken together, these data showed that disruptions in lipid peroxidation and iron homeostasis in alveolar macrophages drive ferroptosis, which is involved in lung inflammation, destruction and remodeling in COPD and can be regulated as a potential target.

## Discussion

**Figure 8:**
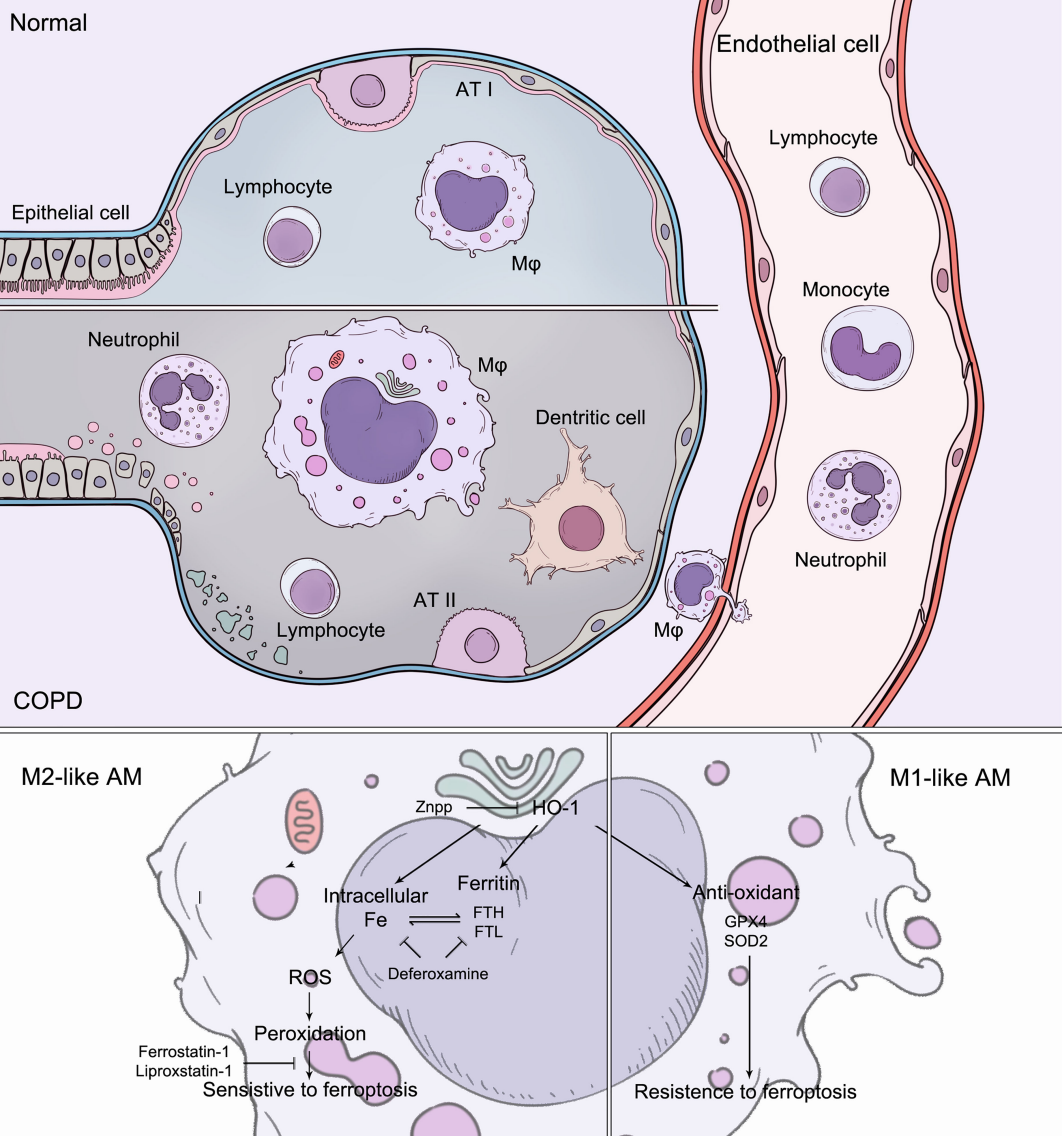
During COPD, the accumulation of lipid peroxidation drives ferroptosis-sensitive M2-like AMs, while M1-like AMs show characteristics of ferroptosis resistance. Increased HO-1 expression in alveolar macrophages modulates susceptibility to ferroptosis. The ferroptotic phenotypes were regulated by anti-ferroptotic compounds, iron chelators, and HO-1 inhibitors.

In this study, we demonstrated that ferroptosis was involved in inflammation in alveolar macrophages in COPD. In alveolar macrophages, there were discrepancies in sensitivity to ferroptosis, which can be determined and regulated by HO-1. During COPD, the accumulation of lipid peroxidation drives ferroptosis-sensitive M2-like AMs, while M1-like AMs show characteristics of ferroptosis resistance. Differential HO-1 expression in alveolar macrophages modulates susceptibility to ferroptosis. This ferroptotic phenotype was ameliorated by anti-ferroptotic compounds, iron chelators, and HO-1 inhibitors, which alleviated lung inflammation and the destruction and remodeling of COPD, representing a potential target (**Figure 8**).

Chronic airway inflammation and lung destruction are critical components of COPD pathogenesis (16). Ferroptosis was identified as a type of necrotic regulated cell death characterized by free iron-dependent phospholipid peroxidation of cell membranes, which is negatively regulated by the selenoprotein GPx4 (40, 41). A recent study demonstrated that epithelial cell ferroptosis was involved in the pathogenesis of cigarette smoke-induced COPD (14, 42). Increased iron burden as evidence of increased concentrations of iron and ferritin in BAL in smoker lungs has been reported (14). Mitochondrial dysfunction and endoplasmic reticulum stress are usually observed in the cytoplasm, and ferroptosis occurs in bronchial epithelial cells (43). The increased ferritinophagy mediated by nuclear receptor coactivator 4 (NCOA4) and reduction of glutathione peroxidase 4 (GPX4) led to the accumulation of free iron and lipid peroxidation during CS exposure. Moreover, GPX4+/− mice showed significantly higher degrees of lipid peroxidation and an enhanced COPD phenotype than wild-type mice, whereas these phenotypes could be attenuated in GPX-transgenic mice (14, 44). PM2.5 is another risk factor for COPD. Increased cellular iron content and ROS production in human endothelial cells were observed after inhaling PM2.5 particles, while the levels of glutathione (GSH) and nicotinamide adenine dinucleotide phosphate (NADPH) decreased. Iron overload and redox imbalance caused by TFRC and ferritin dysregulation are the major inducers of ferroptosis (45). The abovementioned investigations indicated that ferroptosis is involved and plays a crucial damaging role in COPD (40), and searching for an accurate inhibitor of ferroptosis to delay the progression and prevent the occurrence of COPD is pivotal to forthcoming research. Experimental interventions, such as the iron chelator deferoxamine, the ferroptosis inhibitor ferrostatin-1, and suppression of lipid peroxidation by GPX4, could effectively reduce lipid peroxidation, upregulate GSH and NADPH levels, and inhibit ferroptosis (14, 41, 46, 47). Moreover, recent reports revealed that antioxidants, such as N-acetyl-l-cysteine (NAC) and curcumin, could improve the reduction of GSH and reduce lipid peroxidation (46), while dihydroquercetin could inhibit ferroptosis in lung epithelial cells by activating the Nrf2-mediated pathway (48).

Macrophages can be divided into two main phenotypes: M1 and M2. In response to inflammatory signals, such as IFN-γ, LPS and GM-CSF, macrophages polarize into activated M1 macrophages with characteristic transcriptional and secretory profiles, including strong upregulation of iNOS and proinflammatory signals, such as IL-12, IL-1β, and TNF-α. In contrast, alternatively activated M2 macrophages are polarized by anti-inflammatory signals such as IL-4, IL-13 and M-CSF, upregulate genes such as *Arg1*, *Mrc-1* (*Cd206*) and *Cd163*, and release IL-10 (49, 50). Similarly, M1 AMs can produce high levels of proinflammatory and cytotoxic mediators that hinder lung repair, while M2 AMs generate protective factors and orchestrate restorative processes that are beneficial for tissue recovery after injury (51). It has been reported that iNOS-derived NO• can interact with 15-LOX-generated lipid intermediates and is thus a regulator of ferroptotic death in macrophages and microglia *in vitro* (52). In our study, M2 macrophages displayed high sensitivity to ferroptosis, which can be explained by the lack of *Stat-1* and *Stat-2*. Lipid peroxidation has been recognized as a fundamental part of ferroptosis, and it can be divided between two major Fe-dependent pathways: a. nonenzymatic random free-radical chemical reactions (53) and b. a highly selective and specific enzymatic LOX-controlled process (54). The accumulation of lipids and free iron can increase lipid peroxidation in both ways. Given the high expression of ALOX5, enzymatic LOX-controlled reactions are, in our view, most likely prominent in this process.

AM-CLST14-IL1β was characterized by high expression of MHC class II molecules (*Hla-dr*, *Hla-dq* and *Hla-dp*) and antigen processing and presentation (*Cd74*). They also specifically expressed *Il-1β*, *Il-18* and *Tnf*, which are usually recognized as hallmarks of M1 macrophages; however, they also express *Mrc-1* (*Cd206*) and the intermediate *Cd163*. We hypothesized that this cluster of macrophages underwent proinflammatory M1-like polarization. Notably, this cluster expressed high levels of superoxide dismutase (*Sod2*) and glutathione peroxidases (*Gpx1* and *Gpx4*). The *Sod2* gene encodes the mitochondrial antioxidant manganese superoxide dismutase (Mn-SOD), which converts toxic superoxide radicals into less damaging hydrogen peroxide. In previous findings, we observed a clear reduction in *Sod2* between COPD and non-COPD, presumably reflecting increased oxidative stress consumption during disease (55). Glutathione peroxidases (Gpxs) can catalyze the reduction of hydrogen peroxides and lipid hydroperoxides by glutathione to prevent oxidative stress (2). The development and progression of COPD has been strongly associated not only with enhanced ROS production but also with reduced antioxidant capacity. In addition, they expressed intermediate levels of *Ftl*, *Fth1* and *Trfc*, with low levels of *Acsl1* and *Slc3a2*, implying that M1-like AMs were less susceptible to ferroptosis than M2-like AMs.

The presence of foamy AMs (foam cells) is frequently observed in patients with COPD and smokers. Studies have reported that accumulated lipids in AMs are associated with driving lung inflammation (21). It is widely accepted that foam cells derived from lipid-accumulating macrophages drive inflammatory processes in plaques in atherosclerosis (56). Our observations confirmed that the initiation and progression associated with COPD and atherosclerosis (AS) may share similar inflammatory molecular mechanisms. Lung-resident AMs were laden with abundant intracellular lipids, especially very low-density and low-density lipoproteins (VLDL/LDL), and were incapable of dealing with them. This could be explained by the dysregulation of transport proteins (**Figure S3**). This type of AM also expressed high levels of genes mediating innate immune responses, indicating that they initiate an inflammatory cascade resulting in the recruitment of immune inflammatory cells. Lipid-loaded AMs may drive inflammatory processes, similar to pathogenic macrophages in plaques in AS. The source of lipids that accumulate in macrophages is currently not explicit. Given the abundance of phospholipids in surfactant due to hyperresponsiveness in COPD and the critical role that alveolar macrophages play in surfactant regulation, damaged surfactant is the most likely source.

According to reports, alveolar macrophages from patients with COPD show reduced phagocytic uptake of bacteria, such as *Haemophilus influenzae* or *Streptococcus pneumoniae* (57). A high prevalence of bacterial colonization in the lower airways, accounting for over 50% of patients with COPD, can predispose patients to increased infection, inflammatory response and acute exacerbation. Macrophages from patients with COPD are also defective in taking up apoptotic cells, which might contribute to constant inflammation in patients. The mechanism of the defect in phagocytosis appears to be due to defects in scavenger receptors and cytoskeleton organization, which are required for phagocytosis. Another major function of AMs is digesting particles, including exogenous or autologous bacteria, lipids and proteins, via lysosomes. The AMs from COPD patients showed abnormal lysosomes. This does not appear to be a generalized defect, and it may account for decreased lysosomal membrane proteins and some H^+^-transporting ATPases. The cathepsins, on the other hand, were released from AMs to destroy parenchymal construction and drive the immune response (**Figure S4**).

To determine the feasibility of transcriptomic profiling in scRNA-Seq, we examined lung tissue samples obtained from patients with severe COPD (GOLD stage III). We identified a higher proportion of M2-like AMs containing free iron and expressing increased FTH1 and reduced GPX4. In addition, these cells also expressed APOC1, APOE and OLR1. This illustrated that lipid and iron homeostasis are correlated with the disease status of COPD. The patient also had complications of left coronary atherosclerosis and type 2 diabetes mellitus, reflecting the concurrence of diabetes and cardiovascular disease in patients with airway obstruction, which may be related to the deterioration of homeostasis. In another case we included, the patient was identified as having a prominent M2-like AM population but was graded as having moderate COPD. Such patients, in our view, should be followed up more often in cases of acute exacerbation and rapid progression. Thus, the analysis of AMs is promising from a clinical perspective. Although peripheral blood cells are safe and easy to collect and monitor, they have not generated accurate transcriptomic information on disease development and outcome, particularly at the early stage. Alveolar macrophages can be identified using scRNA-Seq or histology analysis to predict and manage the disease; however, repeatedly acquired from bronchoscopic alveolar lavage or biopsy is invasive and intolerable. Sputum specimens containing alveolar macrophages may appear to be an ideal noninvasive way to acquire specimens in practice.

The HO-1 reaction may either exhibit cytoprotection by converting prooxidant hemoproteins and heme to the antioxidants bilirubin and biliverdin or, conversely, exacerbate oxidative stress by releasing ferrous iron and CO. Suppression of HO-1 is related to diseases including obesity, metabolic syndrome and vascular disease (30, 58–60). However, several recent reports have revealed that the role of Nrf2/HO-1 in ferroptosis is controversial due to its antiferroptotic/protective or ferroptotic role in various *in vitro* models, such as *in vivo* and *in vitro* models (35, 61). Using the HO-1 inhibitor ZnPP has been shown to significantly rescue ferroptotic dysfunction of alveolar macrophages and subsequently prevent airway inflammation. The classification of AMs provides clues for developing novel immunotherapy drugs based on the cell populations and their effects. Inhibitors aimed at intracellular free iron (deferoxamine, DFO), lipid peroxidation and ferroptosis (ferrostatin-1 (Fer-1) and liproxstatin-1) and HO-1 may be employed to attenuate the disease. In addition, by examining genes, we found not only currently pursued immunotherapy targets (such as PPARG or PCG-1 activators, e.g., rosiglitazone and pioglitazone) (62) but also other distinct expressions that may serve as targets in the same categories in our dataset. For instance, statins, including simvastatin and rosuvastatin, work by lowering the production of cholesterol and low-density lipoprotein, as well as triglycerides. It can block the metabolic pathway of mevalonate by competitive inhibition of the endogenous rate-limiting enzyme HMG CoA reductase to reduce the synthesis of cholesterol in cells. Statins also stimulate the uptake of apoptotic cells by AMs via inhibition of the prenylation and activation of RhoA (63), which were weakened in M2-like AMs in our data (**Figure S3**). Furthermore, antioxidants such as vitamin E (α-tocopherol), a specific inhibitor of LOX enzyme activity, can increase GSH levels and reduce lipid peroxidation (64–66). Meanwhile, the treatment of comorbidities should be evaluated carefully. For example, studies have shown that anemia was observed in 15-30% of COPD patients, particularly in patients with severe disease, and may be an independent predictor of mortality (67, 68). Sarcopenia/skeletal muscle dysfunction is also an important comorbidity in patients with COPD and is associated with poor quality of life and reduced survival. The prevalence of skeletal muscle dysfunction in patients with stable COPD ranged from 14.5% to 55%. However, iron supplements, nutritional supplements or iron-rich diets should be utilized with caution because they are likely to be detrimental, as iron may increase ROS and ferroptosis. In particular, ferroptosis has been demonstrated to be involved in the pathogenesis of COPD-related skeletal muscle dysfunction (69). Iron levels of COPD patients should be determined, and according to their levels, patients should be supplemented with iron as appropriate. Soluble transferrin receptor (sTfr) reflects iron utilization in the body and can better describe functional or absolute iron deficiency in the body than ferritin and transferrin saturation in clinical practice. High-dose and systemic administration of medications may have adverse effects; thus, it is wise to deliver drugs by the inhaled route.

Taken together, these findings suggest that alterations in iron homeostasis in AMs and discrepancies in sensitivity to ferroptosis play newly discovered roles in COPD pathogenesis. Ferroptotic M2 AMs lose their anti-inflammatory and repair functions but provoke inflammatory responses, resulting in consistent inflammation and tissue damage in the presence of M1 AMs in COPD. We have also demonstrated that the applications of ferroptosis inhibitor, HO-1 inhibitor and iron chelator prevent airway inflammation and lung destruction in COPD. The mechanisms of these inhibitors should be deeply investigated in the future, and further experimental studies and clinical trials may be warranted to test the efficacy of these compounds. Appropriate therapeutic strategies specifically targeting ferroptosis can reduce the occurrence of infections and acute onset and delay the COPD process.

## Data availability statement

The datasets presented in this study can be found in online repositories. The names of the repository/repositories and accession number(s) can be found below: GSE227691 (GEO).

## Ethics statement

The studies involving human participants were reviewed and approved by Ethics Committee of West China Hospital of Sichuan University. The patients/participants provided their written informed consent to participate in this study. The animal study was reviewed and approved by Institutional Animal Care and Use Committee (IACUC) and Animal Experiment Center of Sichuan University.

## Author contributions

YL assisted in conducting experiments, collecting data and writing the manuscript. YY and TG assisted in conducting the experiments. CW assisted in making illustrations. YFY assisted in sample collection. ZW and LZ assisted in designing the study design, conducting experiments, and collecting and analyzing the data. WL designed the study design and supervised and assisted in writing the manuscript. All authors contributed to the article and approved the submitted version.
