# Supplementary material for: Heme oxygenase-1 determines the cell fate of ferroptotic death of alveolar macrophages in COPD
Source: Front Immunol. 2023 May 5;14:1162087. doi: 10.3389/fimmu.2023.1162087 (PMC10196003; doi:10.3389/fimmu.2023.1162087)
Supplement: Supplementary file 1 [file DataSheet_1.docx]

**Supplementary results**

**Lipid accumulation in M2-like alveolar macrophages in COPD**

A heatmap for representative differentially expressed genes from the observed AM population in the three groups is shown (Figure S4A). As described before, the identified transcripts were involved in lipid metabolism and biosynthetic and catabolic processes. We then examined paraffin-embedded lung tissues from twenty-five patients (non-COPD: n=10; mild COPD: n=7, moderate COPD: n=8). The nuclear transcription factor *Pparg* and its [associated](http://xueshu.baidu.com/usercenter/paper/show?paperid=ca36c2cfd04117b75443ad68f2ffea75&site=xueshu_se) pathway were found to be unregulated: *Pparg* was downregulated in samples from mild and moderate COPD, followed by an increase in *Fabp3*, *Fabp4*, *Apoc-1*, *Apoe*, *Me1*, *Olr1*, *Scd2*, *Lpl*, *Scp2* and *Ubc* and a reduction in the expression of genes related to transport and metabolism, such as *Pltp* and *Cyp27a1* (Figure S3A). To validate the heterogeneity of AMs in COPD that we observed in the scRNA-Seq data, we performed Western blot and immunofluorescence examination. Using selective marker genes, we were able to confirm the increase in APOE and CD68 double-positive AMs in patients with COPD (Figure S3B and S3C). Moreover, OLR1-positive AMs were not only increased but also infiltrated into the subendothelium (Figure S3D). We found that the expression of OLR1 increased using Western blotting, which was consistent with the results (Figure S3E).

**M2-like alveolar macrophages showed reduced phagosome and lysosome functions**

The M2-like alveolar macrophages also showed impaired lysosome and phagosome functions: scavenger receptors *Marco* and *Sra1* and the RAS protein activator like 2 (*Rasal2*), RAS association domain family 4 (*Rassf4*), *Rac-1* and *RhoA* were downregulated to some extent in COPD patients, indicating that the reduced scavenger receptors and cytoskeleton rearrangements were involved in weakened phagosome functions (Figure S4B, 4G, 4H). The expression levels of the lysosome-associated membrane proteins *Lamp1* and *Lamp2* and the H^+^-ATPase *Atp6ap1* were also reduced (Figure S4C); in contrast, the expression of CTSD, a lysosomal aspartic protease, was upregulated at both the transcriptional and translational levels (Figure S4E). The higher activity level correlates with a higher content of cathepsin D protein, as observed by qRT‒PCR, Western blot and immunofluorescence (Figure S4E, 4H). Selected networks showed that *α-Tubulin* (*Tuba1b* and *Tuba1c*) and *Rac-1*/*RhoA*, indicating that microtubule instability and actin cytoskeleton rearrangements may underlie the observed cellular phenotypes (Figure S4D).

**Table S1. Patient information**

|  | | | | | | | | | | | |  |  |
| --- | --- | --- | --- | --- | --- | --- | --- | --- | --- | --- | --- | --- | --- |
| Parameter | Smokers without COPD(n=4) | | | | COPD Ⅰ (n=4) | | | | COPD Ⅱ (n=4) | | | | Total |
| Index | C1 | C2 | C3 | C4 | N1 | N2 | N3 | N4 | S1 | S2 | S3 | S4 |  |
| Gender | M | M | M | M | M | M | M | M | M | M | M | M |  |
| Age, yr | 63 | 53 | 64 | 58 | 52 | 76 | 67 | 61 | 70 | 80 | 61 | 78 |  |
| Average Age | 59.50±4.39 | | | | 64.00±8.75 | | | | 72.25±7.50 | | | | 65.25±8.86 |
| BMI | 24.24 | 20.7 | 24.68 | 25.39 | 22.04 | 24.92 | 22.5 | 27.73 | 23.53 | 22.86 | 25.65 | 25.1 |  |
| Average BMI | 23.75±1.80 | | | | 24.30±2.26 | | | | 24.29±1.13 | | | | 24.11±1.81 |
| Smoking, pc×yrs | 40 | 30 | 40 | 20 | 30 | 40 | 80 | 20 | 50 | 50 | 15 | 40 |  |
| Average Smoking | 32.50±8.29 | | | | 42.50±22.77 | | | | 38.75±14.30 | | | | 37.92±16.77 |
| Stage | NA | NA | NA | NA | Ⅰ | Ⅰ | Ⅰ | Ⅰ | Ⅱ | Ⅱ | Ⅱ | Ⅱ |  |
| FEV1 % | 94.7 | 104.5 | 88.7 | 87 | 97.3 | 112.7 | 92.9 | 109.4 | 78.7 | 62.6 | 70.5 | 76.8 |  |
| Average FEV1% | 93.73±6.85 | | | | 103.08±8.21 | | | | 72.15±6.29 | | | | 89.65±14.80 |
| FEV1/FVC % | 92.24 | 82.4 | 79.83 | 83.69 | 69.65 | 69.56 | 60.29 | 68.97 | 69.93 | 56.02 | 69.95 | 57.58 |  |
| Average FEV1/FVC % | 84.54±4.66 | | | | 67.12±3.95 | | | | 63.37±6.59 | | | | 71.68±10.58 |
| BMI: body mass index; COPD: chronic obstructive pulmonary disease; FEV1 %: percent of predicted forced expiratory volume in 1 second. FEV1/FVC %: FEV1 as percentage of forced vital capacity. | | | | | | | | | | | | | |

**Table S2. Primers for qRT‒PCR**

| Gene | Forward | Reverse |
| --- | --- | --- |
| ACSL1 | CACAAGCGGACCAGAGA | GCTCCAACAGAAACCACAG |
| ALOX5AP | TCTCTGGGGAGCCTGAA | CTGGGTCCTGCTTTCGT |
| APOC1 | GACCTCCCAACCAAGCC | TCGACAGAACCACCACCA |
| APOC3 | GGAGCACCGTTAAGGACAA | CGCAGGATGGATAGGCA |
| APOE | TGAGCAGGTGCAGGAGGA | CCACCGGGGTCAGTTGTT |
| ATP6AP1 | CTGTGGCCTGAGAAGGAAG | AACAACCAAGCACCCCAT |
| ATP6V0B | GCTTTGATGTGGCATGGTT | ATGATGGAGGAGCCGGTA |
| ATP6V0D1 | TCCCTGACCTGAGAACCC | GCTGAGGAGCAACATCCC |
| B-ACTIN | GTGGCCGAGGACTTTGATTG | CCTGTAACAACGCATCTCATATT |
| CYBB | CTTCTTCATTGGCCTTGC | CTGAGGGATTGGGCATT |
| CYP27A1 | CGTCAGATCCATCGGGTT | CATCCAGGTATCGCTTCCA |
| FABP3 | GACGGGCAAGAGACCAC | TCAGCAACAGTGCAGTCAG |
| FABP4 | AGCACCCTCCTGAAAACTG | GCAAAGCCCACTCCTACTT |
| FTH1 | AGTCGTCGGGGTTTCCT | GAGGGTGCGGTGAAGAG |
| FTL | GCCAACCAACCATGAGC | GCGGTCGAAATAGAAGCC |
| GAPDH | GCCTCAAGATCATCAGCAA | GTCCTTCCACGATACCAAAG |
| GPX4 | TTGCCGCCTACTGAAGC | ATGTGCCCGTCGATGTC |
| HMOX1 | CAGTCTTCGCCCCTGTCT | GCATGGCTGGTGTGTAGG |
| IL3RA | CAACCCACCATTCTCCAC | TGCAGCTCAAGAAATCCAC |
| KLK15 | TGACAACACCACCAAGCC | TCAGGGGCACAGGAGATAG |
| LAMP1 | GTCCTCATCGTCCTCATCG | CCCTAAGCCCAGAGAAAGG |
| LAMP2 | CAACCCCAATACAACTCACTC | ATGCTGATGTTCACTTCCTTC |
| LIPA | CTTCCGTCGCCTTCTGT | ATGAGTGCAAACGTGGGT |
| LPL | GCTGGACGGTAACAGGAA | CGGACACTGGGTAATGCT |
| MARCO | TGTCCGTCAGGATTGTCG | GGCCCTTCCTTTGGAGTAA |
| ME1 | TTGCTCTTGGTGTTGTGG | GGATAAAGCCGACCCTCT |
| MT-1X | CCACGCTTTTCATCTGTCCC | AGCAGTTGGGGTCCATTTCG |
| OLR1 | AACAAACTAAGCCAGGTATGC | AGAGTGGGTGGAAAGGAAA |
| PLTP | AAGGCCAAGCCAGTCATC | GACCACGCCTCACATCC |
| PPARG | CGAGAAGGAGAAGCTGTTG | TCAGCGGGAAGGACTTTA |
| RAC1 | TGAATCTGGGCTTATGGG | GAACACATCGGCAATCG |
| RASAL2 | CAGCAATAGCAGCATGGA | TCGTGGCAAAGCAAGAG |
| RASSF4 | GCCTGCTTTCAGCCATC | GGCCTTATTCCTGGTCTCA |
| RHOA | GAAATGGCTACGAGAGCTG | TAACCGCATAAGGGCTGT |
| SCD | GAAGAAGCAAGGGCAAGA | TCTCAAATCCCAGGTCTCA |
| SCP2 | TGGGTATGCTGGAAAAGAA | TTGGAACTGGGAATACGG |
| SLC3A2 | ATCAAGGTGGCGGAAGAC | CAGCCGAGCCAGAAGAG |
| SRA1 | CAGAGGGTTGTAGGGTCGT | CGTCCGGTAGCCTCACT |
| TUBA1B | TGAGGGGATGGAGGAAG | GGCCAAAAGGAATGGATAA |
| UBC | CGTCGCAGCCGGGATTT | CACGAAGATCTGCATTGTCAAG |

**Table S3. Unique genes of each alveolar macrophage cluster**

| Cluster 13 | | | | Cluster 14 | | | | |
| --- | --- | --- | --- | --- | --- | --- | --- | --- |
| CCL18 | RMDN3 | DECR1 | NDUFB2 | IL1B | TMEM70 | RNASET2 | PRR13 | CAMTA1 |
| HLA-DRB5 | ACSL1 | TMSB10 | AP2M1 | CCL3L3 | SLC25A5 | PRAM1 | PPA2 | TBXAS1 |
| NUPR1 | ARL4A | SPATS2L | CTNNA1 | SOD2 | PPP1R15A | UBE2D1 | FUCA2 | PSMB3 |
| RNASE1 | CNIH4 | ARL2 | BST2 | EREG | SDHD | RIPK2 | ITGAX | HSD17B4 |
| INHBA | HSP90B1 | ABHD5 | SDC4 | PHLDA2 | TNF | ITM2B | C1orf54 | SLC25A19 |
| FOLR3 | MPHOSPH6 | COPRS | DYNC1I2 | NFKBIA | SH3BGRL3 | UQCRC1 | KLF10 | ATP1B3 |
| LPL | TMEM251 | NUTF2 | FNDC3B | A2M | COTL1 | BANF1 | PEPD | CMTM6 |
| CES1 | CD300C | MTCH2 | TCF7L2 | CDKN1A | SLAMF8 | PVRL2 | KDM6B | PLEKHO1 |
| MMP19 | LY6E | EMILIN2 | TGOLN2 | BCL2A1 | CD58 | RHBDD2 | CRTAP | TCIRG1 |
| CD59 | IFITM3 | STX7 | HADHB | CPVL | IL18 | NFKBIE | SHTN1 | QSOX1 |
| SCD | SLC3A2 | TMBIM1 | PCBD1 | MARCKS | SLC8A1 | SDCCAG8 | HLA-DOA | NFKBID |
| SDC2 | CHMP5 | POR | FKBP15 | G0S2 | EGR2 | CAMK1 | ATP5C1 | NDUFB5 |
| NEAT1 | ATP5H | FKBP2 | C11orf31 | CLEC10A | TM6SF1 | GBP1 | TPI1 | EIF4E |
| CRIP2 | TCN2 | ACOT7 | GSTP1 | HMGN2 | CYFIP1 | HM13 | PRCP | RNF181 |
| MGST1 | YWHAG | TMED9 | CYSTM1 | STX11 | GPX3 | ARRB2 | SORT1 | ARL6IP1 |
| PDK4 | ABCC3 | ALDH1A1 | GNAI2 | RGS10 | DRAM2 | MYD88 | ACTB | REEP5 |
| CCR1 | ARRDC4 | CNDP2 | LAMTOR5 | PPIF | NR4A1 | BST1 | CAT | AHR |
| AQP3 | ACP2 | ANAPC11 | CD164 | CLEC5A | CD300LF | SMS | DUSP6 | NAPRT |
| NUCB1 | SOAT1 | BLOC1S2 | SHFM1 | ZYX | TMBIM4 | ERP44 | NFKBIZ | MEF2A |
| LHFPL2 | PLEC | PPIA | CALU | ICAM1 | TCEB1 | ATP5J | SKAP2 | SAT1 |
| PSMA7 | CALR | COMMD9 | DYNLRB1 | CALM2 | FAM26F | INSIG1 | DEGS1 | GUSB |
| CD36 | ATP6V1D | MT-ND2 | GOLIM4 | CCRL2 | FGL2 | ATP1B1 | DUSP23 | YBX3 |
| ABCG1 | SERINC2 | UQCRQ | MAN1A1 | NINJ1 | CLEC12A | CHP1 | KDELR2 | C14orf2 |
| C20orf27 | FUCA1 | QKI | C1orf43 | ARL8B | MDH1 | ABHD2 | WARS |  |
| NENF | TFPT | NME1 |  | LAT2 | NCF4 | RNPEP | LSM6 |  |
|  |  |  |  | PHLDA1 | CLEC4A | ESD | HVCN1 |  |

**Supplementary figure legends**

Figure S1. Trajectory analysis of the alveolar macrophage state transition based on pseudotime, disease stage and cluster, state, and representative markers of subsets.

Figure S2. Heatmap showing differentially expressed genes of selected ferroptotic genes expressed in datasets (GSE47460-GPL14550, GSE37768 and GSE52509) and their involved and interacting pathways.

Figure S3. Dysfunction of lipid homeostasis in M2-like alveolar macrophages in COPD.

Figure S4. Reduced phagosome and lysosome functions in M2-like alveolar macrophages in COPD.
