# Supplementary figures and images for: Heme oxygenase-1 determines the cell fate of ferroptotic death of alveolar macrophages in COPD
Source: Front Immunol. 2023 May 5;14:1162087. doi: 10.3389/fimmu.2023.1162087 (PMC10196003; doi:10.3389/fimmu.2023.1162087)

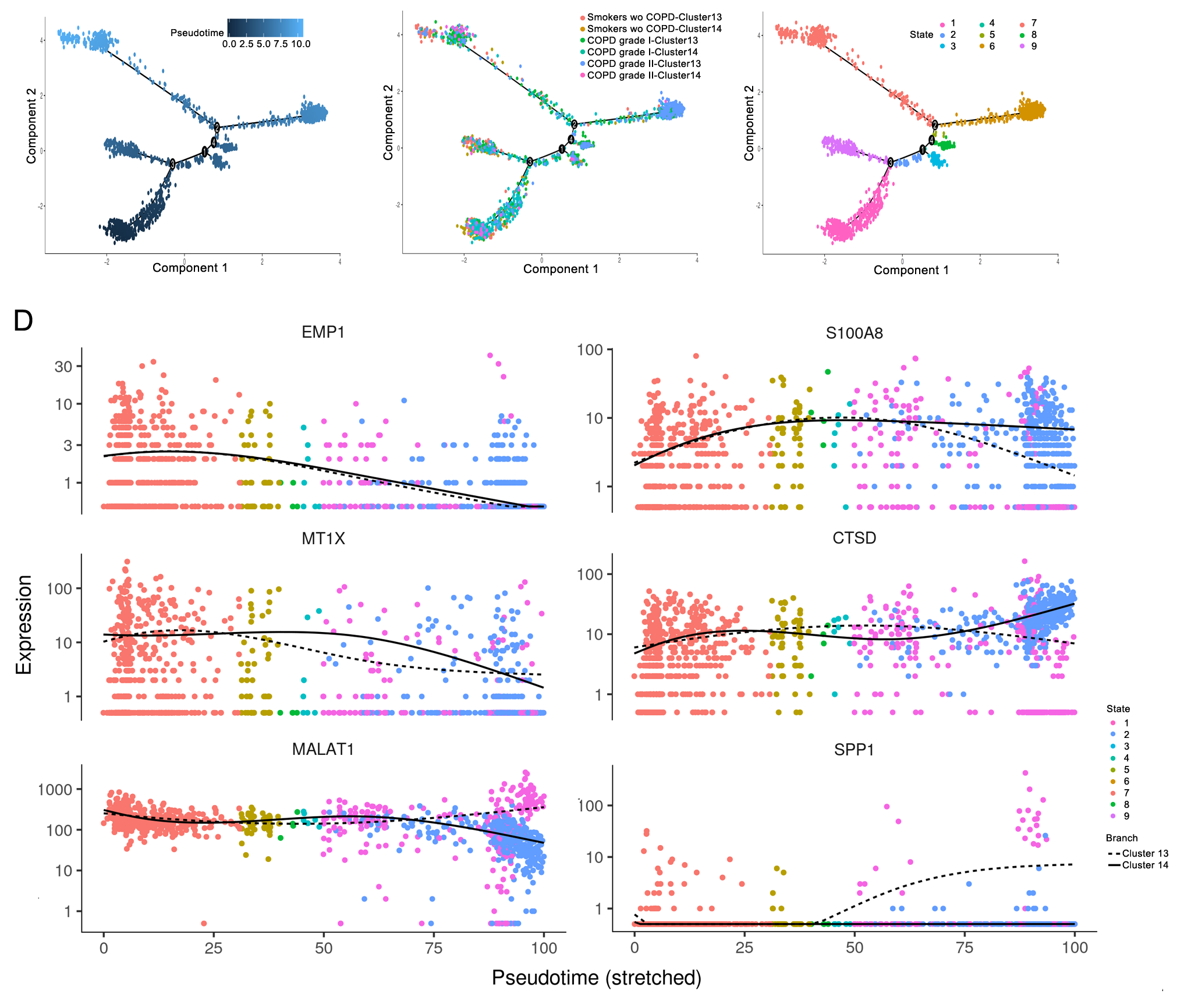

Supplement: Supplementary file 2 [file Image_1.tif]

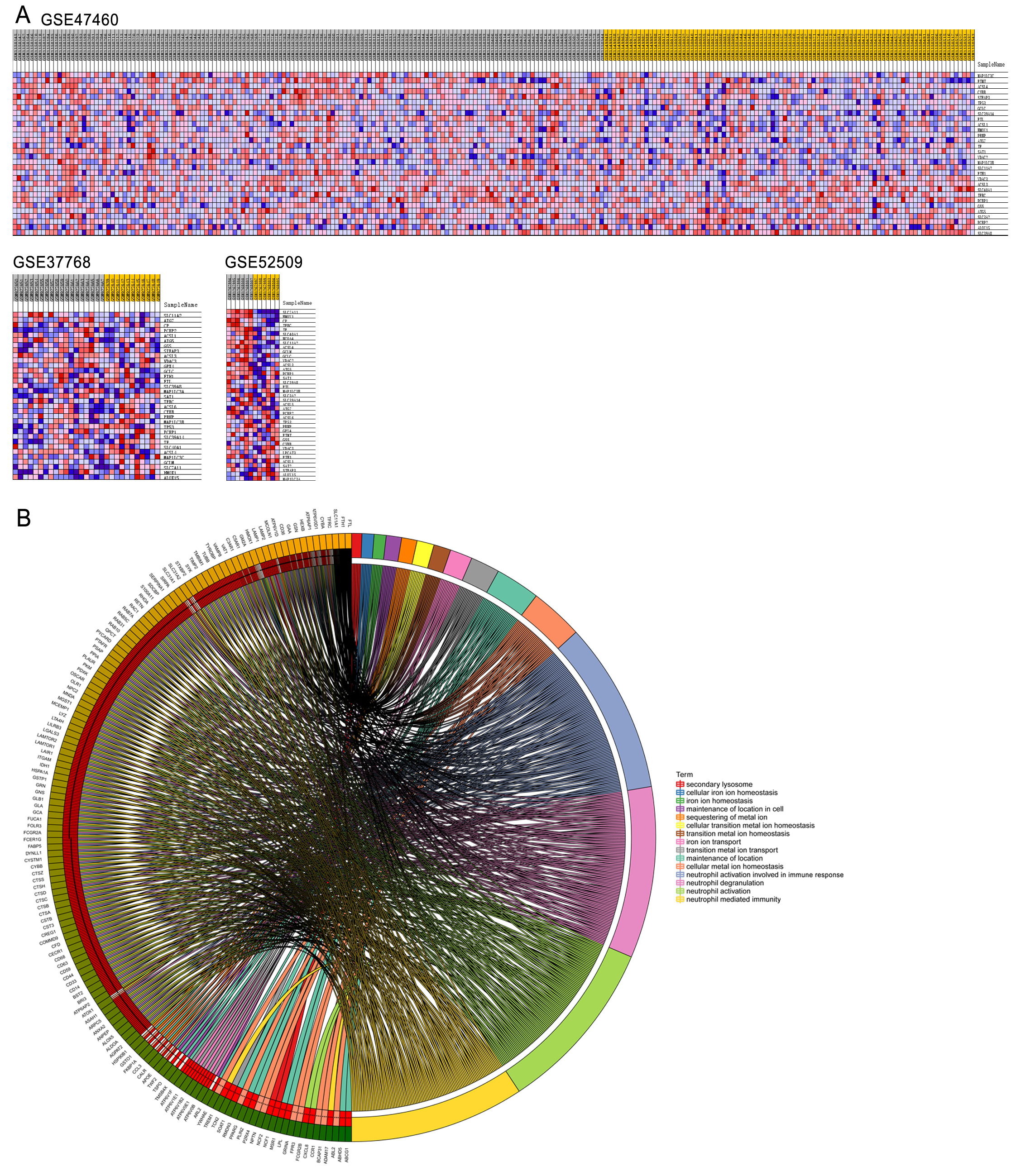

Supplement: Supplementary file 3 [file Image_2.tif]

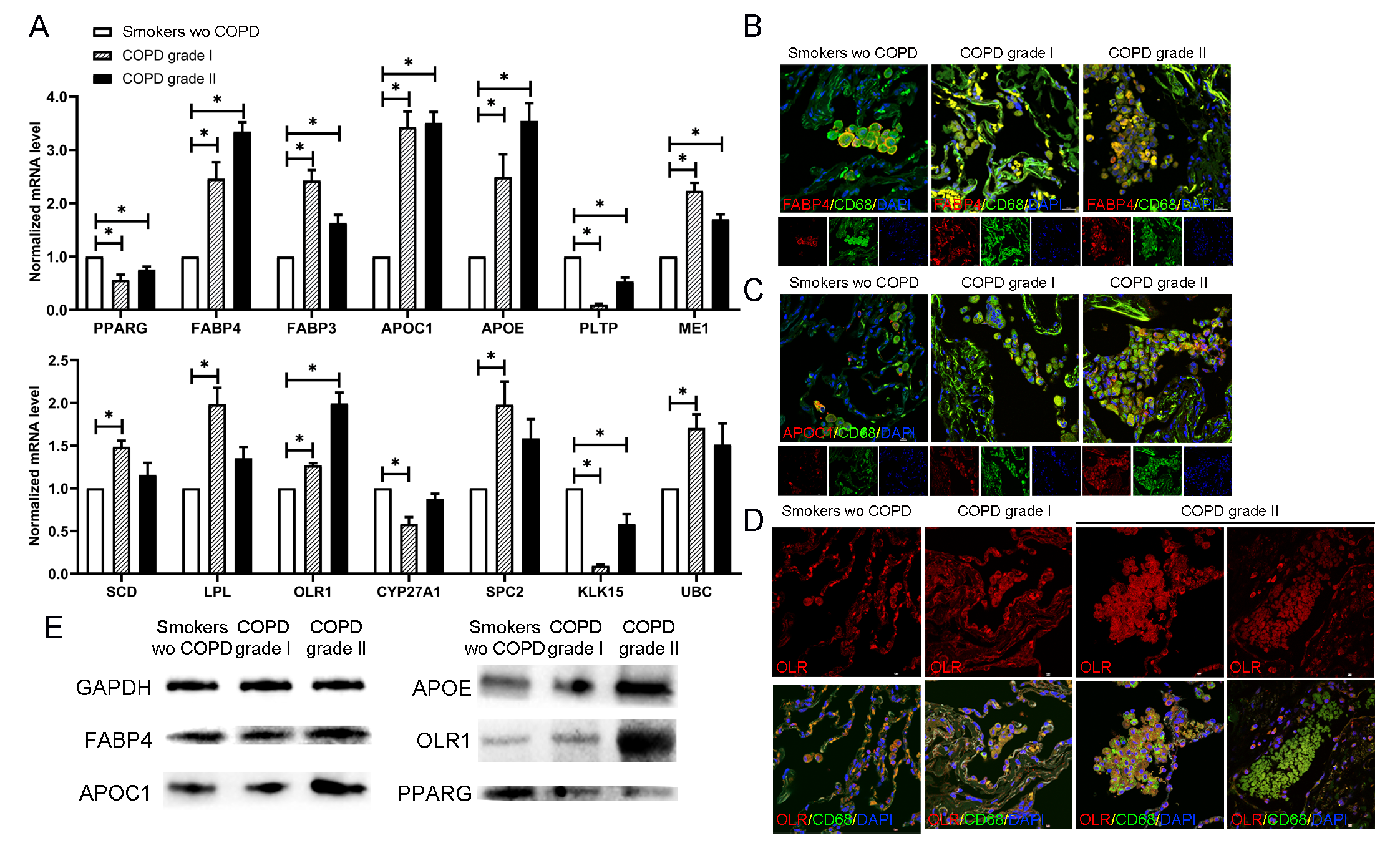

Supplement: Supplementary file 4 [file Image_3.tif]

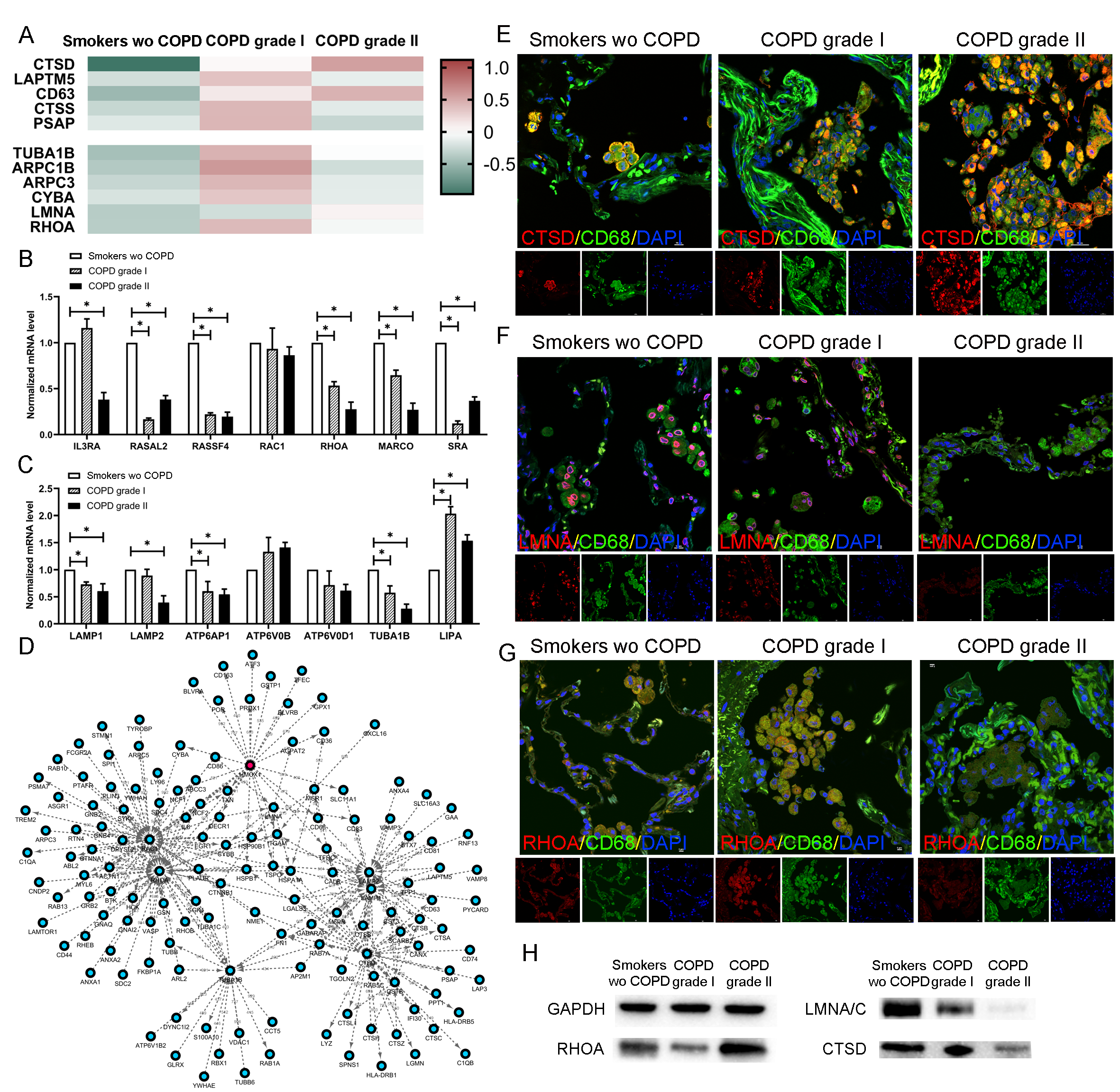

Supplement: Supplementary file 5 [file Image_4.tif]
